# FDG-PET in the evaluation of response to nivolumab in recurrent non-small-cell lung cancer

**DOI:** 10.1186/s12957-016-0998-y

**Published:** 2016-09-05

**Authors:** Mitsunori Higuchi, Yuki Owada, Takuya Inoue, Yuzuru Watanabe, Takumi Yamaura, Mitsuro Fukuhara, Takeo Hasegawa, Hiroyuki Suzuki

**Affiliations:** Department of Chest Surgery, School of Medicine, Fukushima Medical University, 1-Hikarigaoka, Fukushima, 960-1295 Japan

**Keywords:** Positron emission tomography, Nivolumab, Programmed cell death 1 (PD-1), Programmed cell death ligand 1 (PD-L1), Non-small cell lung cancer

## Abstract

**Background:**

Nivolumab, an immune checkpoint inhibitor, is recently clinically applied to non-small cell lung cancer (NSCLC) treatment, and this causes T cell activation and T cell infiltration to tumor tissue through the blockade of the interaction between programmed cell death 1 (PD-1) and programmed cell death ligand 1 (PD-L1). 18F-fluorodeoxyglucose-positron emission tomography (FDG-PET) sometimes shows false positive because of the recruitment of neutrophils, lymphocytes, and macrophages. To date, there is only one report except our case, which described the correlation between FDG-PET and nivolumab.

**Case presentation:**

We report on a 75-year-old man on nivolumab treatment for metastatic non-small cell lung cancer. He had undergone right lower lobectomy for lung adenocarcinoma in the right S8 segment 10 months prior to recurrence. Pathological findings revealed invasive adenocarcinoma, pT1bN2M0 stage IIIA. Epidermal growth factor receptor (EGFR) mutation was positive for de novo T790M and anaplastic lymphoma kinase (ALK) rearrangement was negative. Immunohistochemistry was negative for PD-L1. He underwent chemotherapy with a combination of cisplatin and pemetrexed for four cycles but developed progressive disease involving the right hemithorax, multiple lymph nodes, and multiple osseous sites. Nivolumab was instituted as a second-line chemotherapy. After six courses of this immunotherapy, FDG-PET scan showed decreased FDG uptake in each recurrent lesion despite T lymphocyte activation by nivolumab. Serum carcinoembryonic antigen (CEA) level was also remarkably decreased.

**Conclusions:**

Nivolumab’s effect on recurrent NSCLC may be monitored by PET; larger studies are needed.

## Background

Lung cancer is the leading cause of cancer-related death worldwide [1]. Advances in the treatment of non-smallcell lung cancer (NSCLC) in the past decade include third-generation platinum doublets, epidermal growth factor receptor (EGFR) tyrosine kinase inhibitors (TKIs) in EGFR mutation-positive lung cancer, anaplastic lymphoma kinase (ALK) TKIs in ALK rearrangement-positive disease, maintenance systemic therapy, and second- or third-line treatment, which improve survival [2–9]. In recent years, immune checkpoint inhibitors have been introduced [10–12], and their effectiveness is promising. Nivolumab, an immune checkpoint inhibitor that is an antibody against programmed death-1 (PD-1), causes T cell activation and demonstrates clinically relevant antitumor activity. In some phase III trials, overall survival (OS), objective response rate (ORR), and progression-free survival (PFS) were better with nivolumab than with docetaxel in NSCLC [13, 14].

18F-fluorodeoxyglucose-positron emission tomography (FDG-PET) is an effective tool for assessing treatment response and surveillance of disease recurrence; however, it sometimes shows increased FDG uptake at sites of local inflammatory changes caused by the recruitment of neutrophils, lymphocytes, and macrophages [15]. We present a case of a patient with metastatic NSCLC who underwent nivolumab treatment as a second line chemotherapy and whose PET scan showed decreased FDG uptake after treatment in spite of nivolumab’s known T cell activation properties.

## Case presentation

**Fig. 1:**
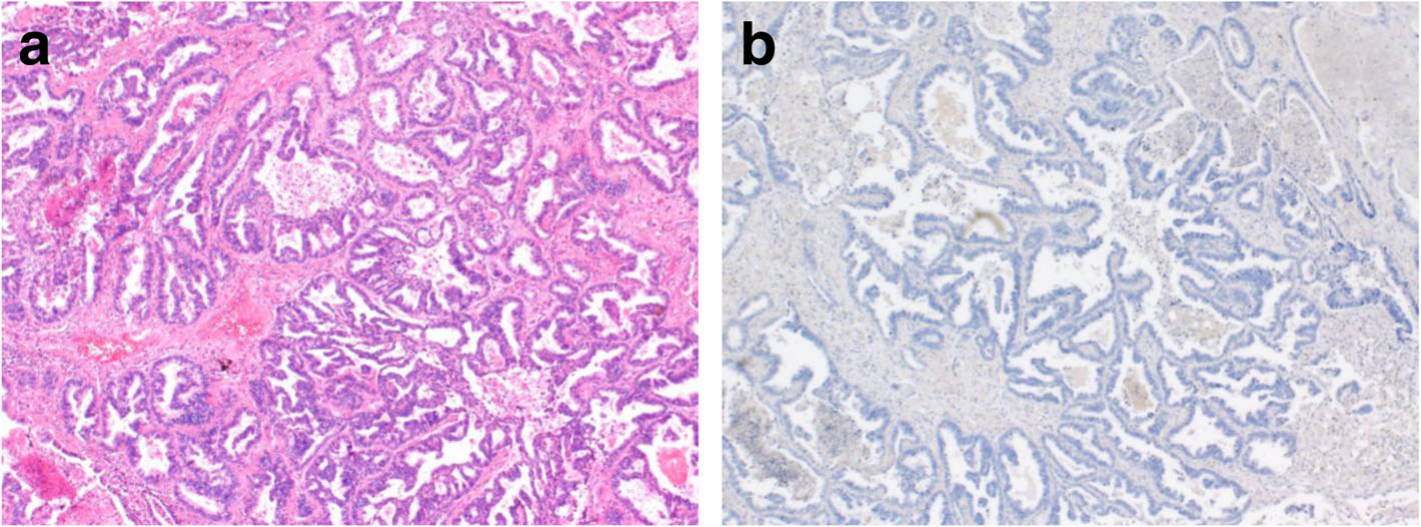
Hematoxylin and eosin (H&E) staining (×100) (**a**) and immunohistochemical staining for PD-L1 (**b**) of surgically resected NSCLC tissue. H&E staining showed invasive adenocarcinoma papillary predominant type and immunohistochemical staining showed negative for PD-L1

A 75-year-old man was admitted to our hospital because of metastatic NSCLC. He had undergone right lower lobectomy for lung adenocarcinoma originating in the right S8 segment as initial treatment. Postoperative findings revealed invasive adenocarcinoma with pT1bN2M0 stage IIIA. An EGFR mutation was positive for de novo T790M, and ALK rearrangement was negative. Immunohistochemistry was negative for programmed death ligand 1 (PD-L1) (Fig. 1). Ten months after his initial surgery, recurrence was detected in the form of multiple bone and lymph nodes metastases and right intrathoracic dissemination. He underwent chemotherapy with a combination of cisplatin and pemetrexed for four cycles (Bevacizumab was withheld because of his past history of cerebral hemorrhage.). After first line chemotherapy, his clinical imaging revealed increasing size and number of metastatic sites, which were graded as progressive disease (PD) according to the Response Evaluation Criteria in Solid Tumors 1.1 (RECIST) [16]. We decided to administer nivolumab (3 mg/kg) every 2 weeks as a second-line chemotherapy. Before starting this immunotherapy, his medical check-up revealed stable borderline diabetes. Hormonal examination such as adrenocorticotropic hormone (ACTH), ftee T3 (FT3), free T4 (FT4), thyroid-stimulating hormone (TSH), and anti-acetylcholine receptor antibody (anti-AchR Ab) were also within normal range. No adverse events occurred during the course of this immunotherapy.

**Fig. 2:**
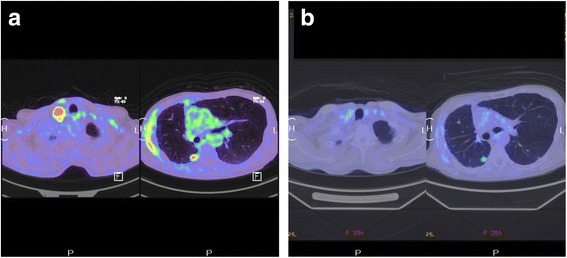
FDG uptake before (**a**) and after (**b**) immunotherapy. Maximum standardized uptake value (SUVmax) of supraclavicular lymph node and disseminated lesions before and after immunotherapy decreased from 9.8 to no accumulation and 5.9 to 3.4, respectively. Each SUVmax was decreased after six courses of nivolumab treatment

**Fig. 3:**
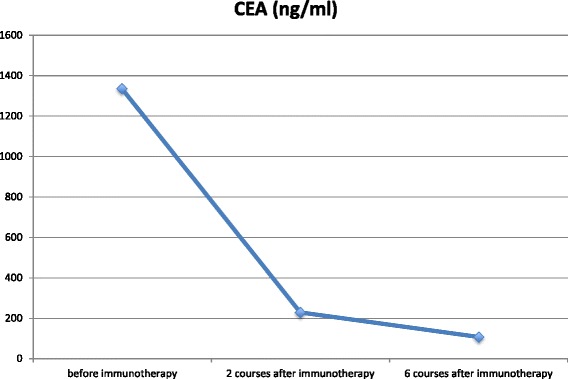
Clinical course of serum carcinoembryonic antigen (CEA) levels during treatment. Serum CEA level decreased from 1335.0 to 108.4 ng/mL after six courses of nivolumab treatment

After six courses of this immunotherapy, an FDG-PET scan showed decreased FDG uptake in each recurrent lesion (Fig. 2). Serum carcinoembryonic antigen (CEA) levels were also decreased (Fig. 3).

### Discussion

In patients with recurrent or advanced NSCLC, who have relapsed after previous platinum-based chemotherapy or EGFR-TKI, docetaxel monotherapy is considered to be the current standard treatment regimen [5, 7]. In a phase III study involving docetaxel in patients with NSCLC previously treated with platinum-based chemotherapy, the time to progression and median survival time (MST) after docetaxel monotherapy has been reported to be 10.6 weeks and 7.0 months, respectively [6]. Recent prospective studies including CheckMate 017 [13] and CheckMate 057 [14] showed that nivolumab was superior to docetaxel for squamous cell carcinoma and non-squamous cell carcinoma previously treated with platinum-based agents. Based on these studies, the current second-line chemotherapy in patients failing platinum-based chemotherapy is considered to be nivolumab. These studies suggested that a radiological response had been detected approximately 8 weeks (4^th^ course) after initial administration of nivolumab.

The combination of FDG-PET and computed tomography (CT) is an effective tool for assessing treatment response and for surveillance for disease recurrence in cases treated with chemotherapy or radiation. Many investigators have reported the utility of FDG-PET or FDG-PET/CT in evaluating therapeutic response prospectively or retrospectively [17–20]. Complete disappearance of abnormal FDG accumulation is an indicator of a low probability of local recurrence and better prognosis after treatment such as surgery, radiation, and chemotherapy. Immunotherapy presents a potential problem because the inflammatory changes caused by the recruitment of neutrophils, lymphocytes, and macrophages [15] may lower the specificity because these cells take up FDG. More specifically, immune checkpoint inhibitors activate T cells, which infiltrate tumor tissue. Uptake by the T cells might affect the evaluation of immunotherapy. To date, there is only one report except our case, which showed the usefulness of FDG-PET/CT to evaluate the efficacy of nivolumab in a patient with indolent Hodgkin lymphoma [21]. Although we usually evaluate the anatomic tumor response with RECIST [16] or immune-related Response Criteria (irRC) [22] after cancer treatment, Wall et al. [23] proposed PET Response Criteria in Solid Tumors version 1.0 (PERCIST) to assess the treatment response instead of RECIST. PERCIST evaluates the metabolic tumor response using the change of SUV ratio in the region of interest between the tumor and liver. The evaluation criteria of PERCIST would be more helpful to evaluate the treatment effects in the cases with FDG-PET.

The optimal follow-up schedule of FDG-PET scan during immunotherapy is unclear. In fact, little is known about the role of FDG-PET and CT scan as appropriate radiological modalities. Some exploratory clinical studies (UMIN000020707 and UMIN000020814) have been conducted and ongoing in Japan to evaluate FDG-PET in nivolumab therapy. In our case, FDG-PET was considered to be a useful tool for the evaluation during immunotherapy. However, it is uncertain what the optimal timing of FDG-PET scanning will yield the highest specificity and sensitivity. More research in this area is needed.

An urgent issue to be resolved is to explore more specific biomarker of this treatment because the correlation between PD-L1 expression by the tumor tissue and treatment effect with nivolumab has not been quantified. However, immunotherapy shows promise to improve the prognosis of patients with unresectable or recurrent NSCLC.

## Conclusions

We have found decreased FDG uptake by tumor after six courses of nivolumab treatment, suggesting the potential value of FDG-PET in monitoring the response to this immunotherapy. T cell activation and infiltration into tumor tissue did not affect the results of FDG-PET in this case. We await detailed results of ongoing clinical studies using FDG-PET for the evaluation of this immunotherapy.

## Abbreviations

ALK: Anaplastic lymphoma kinase
CEA: Carcinoembryonic antigen
CT: Computed tomography
EGFR: Epidermal growth factor receptor
FDG-PET: 18F-fluorodeoxyglucose-positron emission tomography
MST: Median survival time
NSCLC: Non-smallcell lung cancer
ORR: Objective response rate
OS: Overall survival
PD: Progressive disease
PD-1: Programmed death-1
PD-L1: Programmed death ligand 1
PFS: Progression-free survival
RECIST: Response Evaluation Criteria in Solid Tumors 1.1
TKIs: Tyrosine kinase inhibitors
ACTH: Adrenocorticotropic hormone
FT3: Free T3 (free triiodothyronine)
FT4: Free T4 (free tetraiodothyronine)
TSH: Thyroid-stimulating hormone
